# Training and transfer effects of working memory updating training in male abstinent long-term methamphetamine users

**DOI:** 10.1016/j.abrep.2021.100385

**Published:** 2021-10-08

**Authors:** Xin Zhao, Lei Wang, Joseph H.R. Maes

**Affiliations:** a Key Laboratory of Behavioral and Mental Health of Gansu Province, Northwest Normal University, Lanzhou 730070, China; b School of Psychology, Northwest Normal University, Lanzhou 730070, China; c Donders Institute for Brain, Cognition and Behaviour, Centre for Cognition, Radboud University, PO. Box 9104, Nijmegen 6500 HE, the Netherlands

**Keywords:** Methamphetamine addiction, Working memory updating, Training, Transfer effect

## Abstract

Methamphetamine use is associated with cognitive impairments, including executive functioning. These impairments might be cause and/or effect of the drug (re-)use and have, therefore, motivated interventions to improve cognitive functioning. Until now, only very few studies have examined the effect of training working memory updating (WMU), one of the core executive functions, in this population. In the present study, 32 long-term male abstinent methamphetamine inpatients received either a multiple-session WMU training or an active control treatment. All participants performed a number of tasks assessing WMU, inhibition, and task-switching ability- before and after treatment. The WMU-trained patients improved their performance on the trained task and on a non-trained WMU task, reflecting near transfer. However, there was no beneficial training-induced effect for the other tasks, indicating the absence of far transfer. Possible treatment implications of these findings were discussed.

## Highlights

• Abstinent methamphetamine inpatients received working memory updating (WMU) training.
• An active control group of inpatients was included.
• Trained patients showed gain in performance on trained and transfer WMU task.
• There was no evidence of far-transfer effects.
• Results match those of previous WMU training studies with other (non-)addicted samples.

## 1 Introduction

Methamphetamine is a synthetic psychostimulant drug that is widely abused in both western and eastern countries (e.g., National Institute on Drug Abuse, 2021, United Nations Office on Drugs and Crime, 2020). Long-term intake of methamphetamine has multiple negative effects on the brain, health, and daily-life functioning (e.g., Chung et al., 2007, Henry et al., 2010, Homer et al., 2008, Jayanthi et al., 2021, Lappin and Darke, 2021). At least some of the latter two types of effect are mediated by the adverse impact that methamphetamine abuse has on cognitive functioning, including processing speed, language, learning, memory, decision-making, and executive functioning, and underlying brain structures (e.g., Dean et al., 2013, Ellis et al., 2016, Fitzpatrick et al., 2020, Nestor et al., 2011, Potvin et al., 2018, Scott et al., 2007, Weber et al., 2012). These neurocognitive deficits can either be a result of the (ab)use, a predisposing factor for the initial (ab)use or relapse after abstinence, or both (e.g., Basterfield et al., 2019, Lyoo et al., 2015; see also Mizoguchi & Yamada, 2019, for research with rodents).

The question concerning the persistence of these neurocognitive deficits, also as a function of duration of drug use and/or abstinence, is not yet solved. The literature reports mixed, inconclusive research findings. These are also at least partly due to methodological limitations, such as the use of cross-sectional rather than longitudinal studies, and the presence of medical comorbidities and selection biases (e.g., Basterfield et al., 2019, Dean et al., 2013, for reviews, and Scott et al., 2007). However, studies providing evidence for a positive correlation between drug use duration and cognitive deficits and/or for prolonged deficits even after protracted abstinence (e.g., Farhadian et al., 2017, Proebstl et al., 2019) motivate the development of interventions to relieve methamphetamine-induced cognitive deficits.

### 1.1 Improving cognitive functioning

Different types of intervention, aimed at improving cognitive functioning of methamphetamine users, have recently been examined. These include electrical brain stimulation (e.g., Alizadehgoradel et al., 2020), various types of physical exercise (Liu et al., 2021, Menglu et al., 2021, Qi et al., 2021), and cognitive trainings (Bickel et al., 2011, Brooks et al., 2016). The first two types of intervention are promising, but require more systematic research on training parameters and possible modulators of training effects in terms of, for example, duration of drug use and abstinence. However, here we focus on the latter type of intervention: process-based cognitive trainings. More generally, in the field of addiction, various types of cognitive training program have been studied (for reviews, see Sampedro-Piquero et al., 2019, Sofuoglu et al., 2013), but for the purpose of the present study we specifically focus on working memory updating (WMU) training. WMU is one component of executive functions (EFs), a set of higher-order cognitive functions that enable the individual to control cognition and behavior in order to attain his or her goals (e.g., Diamond, 2013). WMU refers to the ability to monitor and update information that is held in working memory and is a core EF (e.g., Diamond, 2016; see also Karr et al., 2018). In some models, the term working memory (WM) is even used more broadly as an overarching concept that includes, next to updating, other aspects of executive functioning like inhibition and cognitive flexibility (e.g., Baddeley & Hitch, 1994). WM and/or WMU are strongly linked to adequate functioning in various daily-life domains, such as academic achievements (e.g., Bergman Nutley & Söderqvist, 2017). Therefore, many studies have examined ways to improve WM/WMU through targeted cognitive training in both healthy and clinical populations. The benefits of such training in terms of positive transfer to performance on non-trained cognitive tasks and daily-life functioning, are moderate and mixed (for reviews, see e.g., Melby-Lervåg et al., 2016, Schwaighofer et al., 2015), and may depend on whether the trained individuals concern healthy adults (benefitting least) or clinical populations (potentially benefitting more), including those with substance use disorders (e.g., Nardo et al., 2021).

### 1.2 WMU training in methamphetamine addiction

To the best of our knowledge, in the context of methamphetamine addiction, effects of WMU training have only been examined in two studies. Brooks et al. (2016) examined 36 polysubstance use inpatients for which methamphetamine was the primary substance of use. The inpatients were assigned to either a treatment as usual (TAU), or a WMU training intervention combined with TAU. The multiple-session WMU training concerned an adaptive *n*-back-task. Before and after the intervention, all participants, as well as healthy controls, completed a number of questionnaires measuring anxiety, depression, mood, impulsivity, and self-regulation, and EF tasks assessing cognitive flexibility and WMU (the latter using the same task as used during training). Moreover, magnetic resonance imaging scans were obtained from all participants. After training, only the WMU-trained group performed more favorably on WMU, self-reported feelings of self-control, and a depression scale. The WMU training group also showed specific changes in volume of frontostriatal brain areas. Brooks et al. (2017) again examined (primary) methamphetamine use inpatients that received either TAU (*n* = 15) or TAU combined with WMU training (*n* = 20), as in Brooks et al. (2016). Before and after treatment, all participants completed self-report measures and a cognitive flexibility task identical to those used by Brooks et al. (2016). A subset of the WMU-trained individuals significantly improved their performance during training. Evidence for a WMU-training-specific benefit was obtained for the mood scale and self-reported feelings of self-control. If anything, the intervention in the TAU group benefitted performance on the cognitive flexibility task more than was the case for the WMU-training plus TAU intervention.

One study (Bickel et al., 2011) assessed the effects of a training program involving WM maintenance, rather than WMU, in 27 individuals with a stimulant abuse diagnosis, 15 for which methamphetamine was the only drug used. Fourteen individuals were assigned to a computerized training program involving various WM tasks (active training group); 13 participants were assigned to a control training condition involving no WM engagement. All participants completed a number of self-report and lab-based instruments to assess WM, different types of inhibition, and episodic and verbal memory before and after the training. The results revealed a training-induced reduction in delay discounting (related to inhibition) for the participants in the active training group only.

The results of these three studies provide some limited evidence for beneficial transfer effects of WM(U) training on executive functioning. However, in the studies by Brooks et al., EFs were assessed using self-report measures, except for cognitive flexibility, and it remains to be seen whether more objective EF tests, also measuring WMU and inhibition, would reveal confirming results. Bickel et al. (2011) did include one WM task and four ‘inhibition’/impulsivity tasks and found positive results for only one of these tasks. However, the number of methamphetamine-only users was very limited (2 and 3 in the control and training group, respectively) and clearly more data are necessary to (dis)confirm these preliminary results using objective tests.

### 1.3 Present study

The present study aimed to add data to the scarce literature on the effect of WMU training in methamphetamine users on their executive functioning, as assessed with common lab tasks. Specifically, we focused on long-term methamphetamine use inpatients that were currently abstinent and undergoing behavioral treatment. One half of the patients was randomly assigned to a common multiple-day WMU training program, whereas the other half was assigned to an active control group. Before and after treatment, all patients were asked to complete EF tasks measuring response inhibition, interference control, WMU, and cognitive flexibility. The question of main interest was whether there would be any significant transfer effects of the WMU training to performance on the untrained EF tasks. Because of the relatively many observations of null results reported in the general cognitive training literature, we performed Bayesian analyses to examine the strength of the evidence for the hypotheses, next to using inferential hypothesis-testing analyses.

## 2 Methods

### 2.1 Participants

Thirty-two male abstinent methamphetamine-dependent inpatients from the Zhejiang Shiliping Drug Rehabilitation Center (Zhejiang province, China) served as participants. The participants had used methamphetamine for 16–26 years and all participants had received a withdrawal treatment 5–7 months before the start of the study. The patients’ present treatment included physical exercise and psychosocial education. The participants were randomly assigned to a training or control group (see Table 1 for the groups’ characteristics, and Supplementary Material for a CONSORT flow diagram). None of the participants had a current or previous history of psychosis, as confirmed by clinical staff and screening questionnaires. None of the patients were using a prescribed medication during the study (e.g. anti-psychotic, anti-depressant, antianxiety medications and/or medications for attention deficit hyperactivity disorders) that might have affected cognitive performance and the effects of WMU training. All participants were fluent in Chinese and literate. According to the admission regulations of the rehabilitation center, the patients were not allowed to use alcohol, cigarettes, or any other addictive substances during their stay in the center. Compliance with this regulation was confirmed by urine tests. None of the participants had participated in a similar study before. All participants voluntarily participated after signing an informed consent form. Ethical approval for the study was obtained from the ethics committee of Northwest Normal University and all experimental manipulations were performed according to the approved guidelines.

**Table 1:** Characteristics of control and trained groups.

	CTRL	TRAIN	p*
N	16	16	
Age	41.1 (3.1)	39.4 (2.7)	0.11
Years of heroin use	20.7 (2.9)	19.8 (3.5)	0.45
Months of abstinence	6.2 (0.8)	6.1 (0.8)	0.83

Note: Values represent means (±standard deviations). CTRL = control group; TRAIN = trained group. *p-value associated with *t*-test examining between-group difference.

### 2.2 Tasks and measures

The training and pre- and post-treatment assessment tasks were identical to those used in the study by Zhao, Wang, and Maes (2020) and will only be briefly outlined here.

#### 2.2.1 Training task

The training task consisted of a visual adaptive *n*-back task. This task (or similar versions of it) have been frequently used in training studies with other populations (e.g., Jaeggi et al., 2010, Studer-Luethi et al., 2016). On each trial, a visual stimulus (a blue square) was presented in one of eight spatial locations of a 3 × 3 matrix for 500 ms; the inter-stimulus interval was 2500 ms. On each trial, the participant had to indicate whether the spatial position of the current stimulus was identical to that of the stimulus presented *n* trials back (a “match”) or not (a “non-match”). The participant had to press the letter A in case of a match, and to refrain from responding on non-match trials. The value of *n* was either decreased, increased, or remained unchanged for each individual participant, based on the participant’s performance accuracy level on preceding trials. Specifically, *n* was increased by 1 in case the accuracy was larger than 90%. The *n* level was decreased if the accuracy was below 70%. Finally, the value of *n* remained unchanged in case of an accuracy level between 70% and 90%. Each participant started with *n* set at two. Each training session lasted about 25 min and consisted of 20 blocks. Within each block, the number of trials was 20 + *n.* The dependent measure was the mean *n*-back level achieved on each training session.

#### 2.2.2 Pre- and post-treatment assessment tasks

##### 2.2.2.1 Interference control tasks

Standard Stroop and flanker tasks were used to assess interference control. In the Stroop task, the participant had to indicate the color of the letters forming a color word as fast and accurately as possible. The color was either congruent or incongruent with the color word. In the flanker task, the orientation of a centrally presented target stimulus (pointing to the right or left) was either congruent or incongruent with the orientation of flanking stimuli. On each trial, the participant had to indicate the orientation of the target stimulus as fast and accurately as possible. The dependent measures for each task was the difference in mean response time (RT) and accuracy for congruent and incongruent trials, with a large difference reflecting poor interference control.

##### 2.2.2.2 Go/no-go (GNG) task

This task was used to assess response inhibition capacity. The letter X or Y was presented on each trial and, in the first half of the task, the participant consistently had to respond to each X (go trials) and not respond to each Y (no-go trials). This was reversed during the second half of the task (letter X now being the no-go letter). The percentage of go and no-go trials was 70%, and 30%, respectively. Due to a programming error, performance on the last block of 100 trials (from the total of 400 trials) was not included in the analysis for eight participants. The performance sores of these participants did not significantly differ from that of the other participants, and inclusion or exclusion of their data did not affect the (null) results. The dependent measure was the difference in proportion of correct responses on go trials (“hits”) and incorrect responses on no-go trials (“false alarms”), with a low score reflecting poor response inhibition.

##### 2.2.2.3 Switching task

Task-switching ability was assessed with a task in which, on each trial, the participant was shown a digit. The participant had to decide whether the digit was larger or smaller than 5 (Task A) or odd or even (Task B), as fast and accurately as possible. On some trial blocks, the task was the same for all trials (single-task trials). Other trial blocks contained both Tasks A and B (mixed-task blocks). Successive trials within mixed-task blocks involved either a task repetition (non-switch trials) or a task switch (switch trials). The dependent measures were the mixing cost (the difference in RT and/or accuracy between non-switch trials from mixed-task blocks and single-task trials) and the switch cost (the difference in RT and/or accuracy between switch and non-switch trials). High mixing and switch costs imply a poor task-switching ability.

##### 2.2.2.4 Running memory (RM) tasks

These tasks were used to assess WMU ability (e.g., Norris & Jones, 1990). Single digits were sequentially presented and the participant had to memorize the last three presented digits. Different sequence lengths were presented and the participant was not informed about the length of the current sequence. The participant had to reproduce the final three digits at the end of each sequence. There were two task variants that differed in presentation time of each digit (either 750 or 1750 ms). The dependent measure for each task variant was the proportion of all to-be-remembered target digits that were correctly reproduced in the correct order. Analysis revealed similar transfer effects for the two task variants. In the Results section, we report the results of an analysis using the mean proportion correct responses based on the two task variants.

### 2.3 Procedure

All participants first performed the Stroop, flanker, GNG, RM, and switching tasks within 1–2 days in a standardized laboratory setting. The patients in the training condition then finished 20 daily training sessions within 20–21 days. Participants in the control condition instead worked on sand paintings (e.g., see Zhao, Chen, & Maes, 2018) at the same times and in a comparable location. All participants again performed the transfer tasks, as during the pre-treatment assessment.

### 2.4 Data analysis

Using JASP 0.14.1.0 (JASP Team, 2020) the training data were subjected to a repeated measures analysis of variance (RM-ANOVA), with session as within-subject factor and mean achieved *n* level as dependent measure. Next, we performed validity checks on the transfer tasks, using RM-ANOVAs and one-sample *t* tests. For the Stroop and flanker tasks, we checked whether incongruent trials elicited longer RTs than congruent trials. Concerning the switching task, we tested whether there was a difference between the RT on single-task, non-switch, and switch trials, with the latter trial type eliciting the longest RT. For the GNG task, we tested whether no-go trials elicited more incorrect responses than go trials. Finally, for the RMT tasks we tested whether the proportion correct responses significantly deviated from 1, to check for possible ceiling effects. All these tests confirmed the validity of the tasks in the present population, *p*s < 0.001 (data not shown). Next, ANOVA was performed on each of the pre-treatment task performance outcome scores, with group as single between-subjects factor, to confirm the absence of significant performance differences between the two groups prior to the differential treatment. Each post-treatment performance outcome score was then subjected to analysis of covariance (ANCOVA), with the corresponding pre-treatment score as covariate. The overall accuracy scores for the Stroop, flanker, and switching tasks were high and there were no signs of speed-accuracy trade-offs. Therefore, for these tasks, we focused on, and only reported, the RT data. We adopted an alpha level of *α* < 0.05 as significance criterion, and partial eta-squared (*η_p_*^2^) as effect size measure, for these inferential hypothesis-testing analyses. We also performed Bayesian ANCOVAs, adopting the following criteria (Van Doorn et al., 2021): a Bayes factor (B_10_) between 1–0.33, 0.33–0.10, and 0.10–0.03 indicates weak, moderate, and strong evidence for the null hypothesis (H_0_), and BF_10_ between 1–3, 3–10, and 10–30 indicates weak, moderate, and strong evidence for the alternative hypothesis (H_1_), respectively. H_0_ represents a model including the intercept and the pre-treatment score (covariate), whereas H_1_ represents a model additionally including the group factor. In a final analysis, we examined whether individual differences in training progress were significantly correlated with individual differences in the transfer benefit that we observed for the RMT (the only task for which we found reliable transfer, see below). For this purpose, for each trained individual, we computed the difference in performance on the last and first training session (training change score), which we correlated with the difference in post- and pre-treatment RMT performance score (RMT change score) using a Spearman correlation analysis.

## 3 Results

### 3.1 Performance across training sessions

**Fig. 1:**
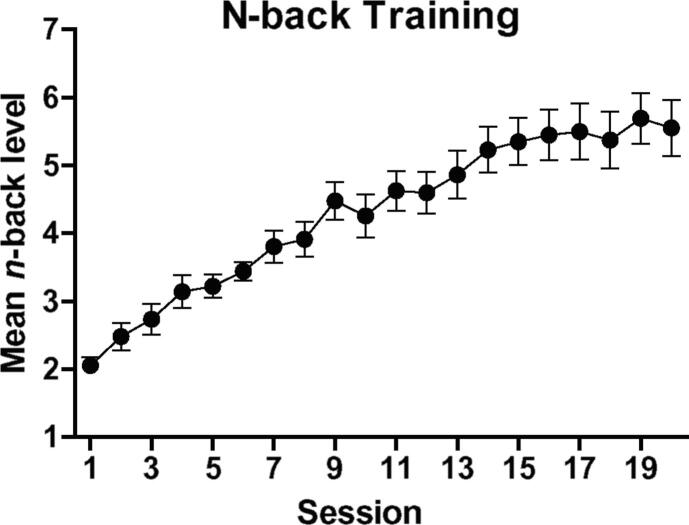
Mean (+standard error of the mean) *n*-back level reached by the patients on each of the 20 training sessions.

Fig. 1 shows the training progress across the 20 training sessions. RM-ANOVA revealed a highly significant effect of session, *F*(19, 285) = 37.18, *p* < .001, *η_p_*^2^ = 0.71, which reflected a significant linear increase, *F*(1, 15) = 81.01, *p* < .001, *η_p_*^2^ = 0.84, and a significant quadratic pattern, *F*(1, 15) = 11.30, *p* = .004, *η_p_*^2^ = 0.43.

### 3.2 Transfer task performance

Table 2 shows the score on each of the outcome measures, separately for each group and assessment session. The two groups did not differ on any of the pre-treatment outcome scores, *F*s < 1.75, *p*s > 0.20, *η_p_*^2^s < 0.06. The table also displays the outcome of the ANCOVAs. The traditional ANCOVAs only revealed significantly better post-treatment performance for the trained patients on the RMT, reflecting near transfer. The Bayesian ANCOVA found strong evidence for the model including the group factor (reflecting better performance for the trained individuals) relative to the null model with respect to the RMT. Weak evidence for the null model was obtained for each of the other performance outcome measures.

**Table 2:** Score on pre- and post-treatment test measures for the trained and control groups, and results of traditional and Bayesian ANCOVAs.

Task/Measure	TRAIN		CTRL		ANCOVA	ANCOVA
	Pre	Post	Pre	Post	*F, p, η_p_^2^*	BF_10_
Stroop-RT diff	29.42 (70.48)	16.30 (44.02)	30.55 (46.86)	21.65 (28.51)	0.17, 0.69, 0.01	0.34
Flanker-RT diff	39.12 (34.99)	15.97 (9.94)	25.23 (23.23)	25.39 (41.55)	1.17, 0.29, 0.04	0.51
GNG-H-FA	0.87 (0.08)	0.92 (0.05)	0.93 (0.05)	0.95 (0.03)	1.63, 0.21, 0.05	0.76
Switching-RT-SC	151.96 (179.31)	125.91 (129.91)	179.96 (188.78)	152.55 (155.96)	0.29, 0.59, 0.01	0.39
Switching-RT-MC	112.28 (127.89)	90.06 (90.53)	110.36 (150.64)	45.83 (104.96)	1.62, 0.21, 0.05	0.63
RMT	0.83 (0.13)	0.94 (0.06)	0.85 (0.14)	0.82 (0.17)	13.96, **0.001**, 0.33	**29.95**

*Note*. Values represent means (and SDs). Pre and Post = pre- and post-treatment assessment. RT diff = difference between mean RT on incongruent and congruent trials. GNG-H-FA = difference between percentage hits and false alarms for the go/no-go task. Switching-RT-SC = RT-based switch cost for the switching task. RT-MC = RT-based mixing cost for the switching task. RMT = proportion correct responses during the running memory tasks. *p* values in bold are significant at *α* = 05. Bayesian factor (BF_10_) in bold corresponds to strong evidence for H_1_.

### 3.3 Relation between training and RMT transfer benefits

For the trained individuals, the magnitude of the training benefit was not significantly related to the magnitude of the pre- to post-training RMT performance benefit, Spearman’s *ρ* = 0.007, *p* = .98.

## 4 Discussion

### 4.1 Summary of findings

This study assessed the effects of a multiple-day, adaptive WMU training program on the executive functioning of long-term, abstinent inpatient methamphetamine users. We found that the patients improved their performance on the trained task. Moreover, relative to patients from an active control group, the trained individuals showed an improvement on an untrained WMU task, reflecting a beneficial near-transfer effect. However, no evidence was found for training-induced benefits with respect to tasks measuring interference control, response inhibition, and cognitive flexibility, reflecting the absence of far-transfer effects.

### 4.2 Integration with previous studies and possible implications

The present results partly confirm the results reported by Brook et al. (2016; 2017), who also found that trained patients were able to improve on the trained WMU task, but did not find any significant benefit for a far-transfer lab task measuring cognitive flexibility. Bickel et al. (2011), training their patients with a program involving tasks primarily tapping memory span rather than WMU, also failed to find transfer to lab-based tasks measuring inhibition (including a GNG task similar to ours). The only transfer they found was to a delay-discounting task, which may be argued to not be a classic, “process pure” EF task. Arguably, the positive results found for the self-report measures in the Brooks and Bickel et al. studies might have reflected demand characteristics elicited by the patient’s enrollment in a “therapy”.

The collective results, suggesting training and near-transfer effects but no objective far-transfer effects of WMU training, strongly mirror those of the majority of studies on the effects of such training in other clinical populations (and healthy participants for that matter; see reviews in introduction, and also Soveri, Antfolk, Karlsson, Salo, & Laine, 2017). In this respect, the present population of methamphetamine users does not seem to fundamentally differ from other clinical populations, including those consisting of (ab)users of other substances. However, the training and near-transfer effects could still have clinical implications and relevance in a more indirect way. In a general sense, they demonstrate a fundamental capability to learn new cognitive tasks and to generalize the newly acquired or strengthened capacity and/or skills to more-or-less similar tasks. In principle, such capacity or skills could also help the individual suffering from a drug use disorder to profit more from (elements of) other types of (standard) treatment, such as psychosocial education. For example, if the training implied an enhanced capacity or learned strategy to better monitor provided information, this could also enhance the effect of educational measures.

The issue of potential benefits of WMU training, and other types of cognitive training, is also strongly linked to the question of what exactly is being learned or changed through such training: enhancement of a, relatively domain-general, WMU capacity, or some relatively task-specific strategy or skill. Arguably, the former would imply stronger expected clinically relevant benefits than the latter. In this framework, it is notable that our study failed to find a direct (linear) association between training and WMU transfer-task improvements. This null result could perhaps be seen as evidence for the strategy hypothesis. Specifically, during training, all participants might have fully learned to adopt a specific strategy that the large majority of the participants could also successfully apply to the WMU transfer task (14 out of the 16 participants showed a pre- to post-training WMU transfer-task performance). Alternatively, the null result could still be interpreted in terms of a training-induced WMU capacity enhancement when assuming a non-linear association between training and transfer-task improvement. Particularly, during training, all participants might have reached, for example somewhere halfway the training, a maximum with respect to the WMU capacity that was necessary to significantly improve performance on the transfer task.

### 4.3 Limitations and future research

A limitation of the present study is the low sample size. This means that the study was underpowered both with respect to the traditional and most of the Bayesian analyses. Concerning the latter, only inconclusive evidence was obtained for the absence of transfer to the Stroop, flanker, GNG, and switching tasks, and more measurements would have been necessary to further enhance this evidence. However, sufficient data were collected to yield strong evidence for a training-induced WMU transfer-task performance benefit. Also given the very low effect sizes revealed by the traditional statistics and the general literature on cognitive training effects, we think it is plausible to assume a real absence of far transfer effects in the present study. A further limitation was the use of only one task to measure response inhibition, WMU, and task-switching, and two tasks to assess interference control. However, our tasks are commonly used and the results are in agreement with the general cognitive training literature (partly using other tasks to measure the different EFs). Another potential limitation concerns discussions about the reliability and/or validity of the *n*-back and RM tasks as tasks tapping WM capacity in general, or WM updating in particular. Concerning the *n*-back task, many studies question at least certain types of validity and/or reliability of the task (e.g., Jaeggi et al., 2010, Redick and Lindsey, 2013). However, other, more recent studies suggest at least acceptable reliability (e.g., test–retest validity: Soveri et al., 2018) and validity (e.g., construct/concurrent validity: Frost et al., 2021). Irrespective of this discussion, the *n*-back task is perhaps the most common experimental WM training paradigm, and we were interested in assessing the effects of this type of WM training in the context of the special population of our study. Regarding the RM task, it is discussed in the literature whether it only measures updating when using relatively long item presentation times (Bunting, Cowan, & Saults, 2006), or whether it involves WM updating at all rather than simple retrieval from a passive storage (Broadway and Engle, 2010, Elosúa and Ruiz, 2008, Palladino and Jarrold, 2008). However, more recent research suggests that performance on this task does involve WM updating when task demands are such that the participant has sufficient resources to adopt a “true” WM updating strategy (Botto, Basso, Ferrari, & Palladino, 2014), although it may not be updating of the item but of item-item associations (e.g., Artuso & Palladino, 2014; further discussion of this topic is outside the scope of this article). Such task demand might be that the participant is given sufficient time to actively process the to-be-remembered items. In this framework, it is noteworthy that our fast-paced version of the task (the RM 750 ms task) implied an item presentation time that was still longer than that used in the fast presentation conditions used by Bunting et al. (2006; 250 ms) and Broadway and Engle (2010; 500 ms). Another limitation is that no follow-up was included in our study so that we do not know how long the beneficial near-transfer effect would last. Finally, we did not include measures of direct clinical relevance, such as self-reports about emotional states, and objective measures concerning relapse. Therefore, it remains to be seen in future research whether the observed near-transfer effect would have any clinical implications. In this framework, such research should also further examine the mechanism underlying the near-transfer effect, specifically whether it reflects a capacity enhancement or the learning of some strategy. Such research could, for example, use more tasks to assess WMU, use parametric variations of training level, and employ (structured) interviews on how the training task was approached.

## 5 Conclusions

Long-term male abstinent methamphetamine use inpatients either received a WMU training or a control treatment. Before and after treatment, all patients were assessed on tasks measuring inhibition, task-switching, and WMU abilities. The WMU-trained participants improved performance on the trained task and showed a training-induced beneficial effect on the non-trained WMU task, reflecting near transfer. However, relative to the control patients, they did not show better post-treatment performance on the other tasks, reflecting the absence of far transfer. These results match those generally found in the cognitive training literature. Possible implications of the patients’ demonstrated capability of learning, and their ability to apply the training-induced changes in capacity or skills for treatment remain to be addressed in future research.

## Declaration of Competing Interest

The authors declare that they have no known competing financial interests or personal relationships that could have appeared to influence the work reported in this paper.

## Data Availability

The data that support the findings of this study are openly available in Mendeley at https://doi.org/10.17632/xwjmd598pv.1. Raw data are available upon request.
