# Supplementary figures and images for: Training and transfer effects of working memory updating training in male abstinent long-term methamphetamine users
Source: Addict Behav Rep. 2021 Oct 8;14:100385. doi: 10.1016/j.abrep.2021.100385 (PMC8664865; doi:10.1016/j.abrep.2021.100385)

CONSORT flow chart


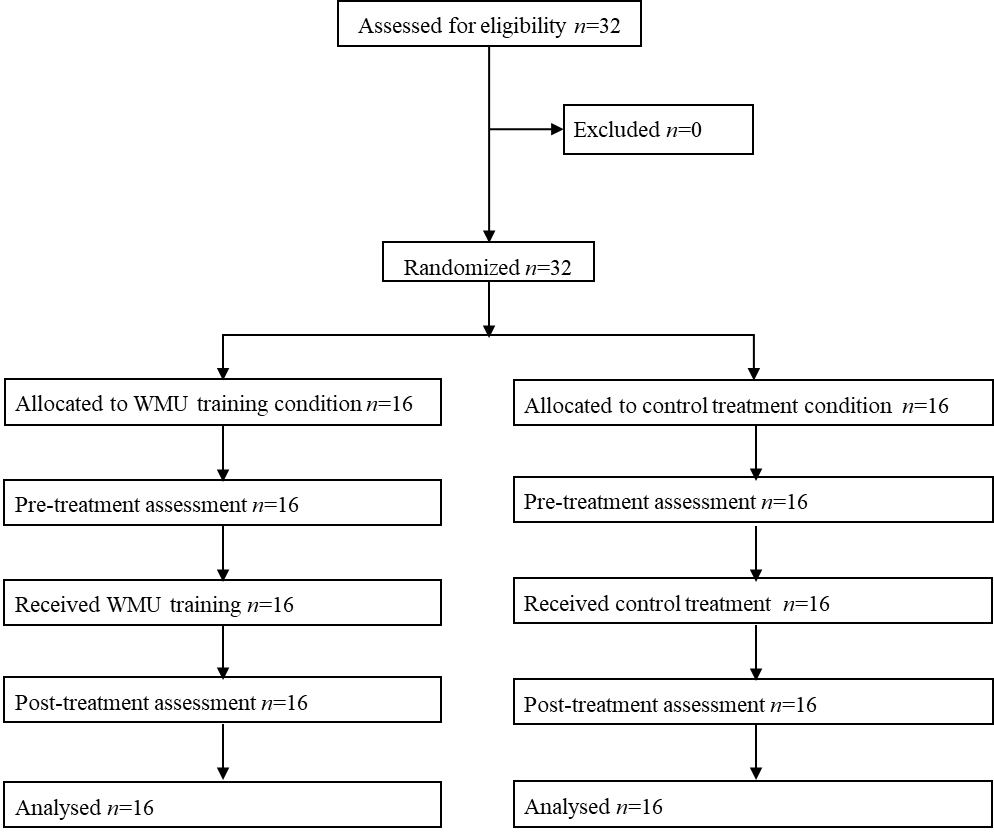

Supplement: Supplementary Data 1 [file mmc1.docx]
